# Biocides and Novel Antimicrobial Agents for the Mitigation of Coronaviruses

**DOI:** 10.3389/fmicb.2020.01351

**Published:** 2020-06-23

**Authors:** Govindaraj Dev Kumar, Abhinav Mishra, Laurel Dunn, Anna Townsend, Ikechukwu Chukwuma Oguadinma, Kelly R. Bright, Charles P. Gerba

**Affiliations:** ^1^Center for Food Safety, The University of Georgia, Griffin, GA, United States; ^2^Department of Food Science and Technology, The University of Georgia, Athens, GA, United States; ^3^Department of Soil, Water and Environmental Science, University of Arizona, Tucson, AZ, United States

**Keywords:** coronavirus, SARS-CoV-2, COVID-19, disinfection, antimicrobial, biocide, mitigation

## Abstract

In December, 2019, a highly infectious and rapidly spreading new pneumonia of unknown cause was reported to the Chinese WHO Country Office. A cluster of these cases had appeared in Wuhan, a city in the Hubei Province of China. These infections were found to be caused by a new coronavirus which was given the name “2019 novel coronavirus” (2019-nCoV). It was later renamed “severe acute respiratory syndrome coronavirus 2,” or SARS-CoV-2 by the International Committee on Taxonomy of Viruses on February 11, 2020. It was named SARS-CoV-2 due to its close genetic similarity to the coronavirus which caused the SARS outbreak in 2002 (SARS-CoV-1). The aim of this review is to provide information, primarily to the food industry, regarding a range of biocides effective in eliminating or reducing the presence of coronaviruses from fomites, skin, oral/nasal mucosa, air, and food contact surfaces. As several EPA approved sanitizers against SARS-CoV-2 are commonly used by food processors, these compounds are primarily discussed as much of the industry already has them on site and is familiar with their application and use. Specifically, we focused on the effects of alcohols, povidone iodine, quaternary ammonium compounds, hydrogen peroxide, sodium hypochlorite (NaOCl), peroxyacetic acid (PAA), chlorine dioxide, ozone, ultraviolet light, metals, and plant-based antimicrobials. This review highlights the differences in the resistance or susceptibility of different strains of coronaviruses, or similar viruses, to these antimicrobial agents.

## Introduction

Coronaviruses, members of the family *Coronaviridae* and subfamily *Coronavirinae*, were initially considered epizoonotic in nature within avian and mammalian hosts (Peeri et al., 2020; Sahin et al., 2020). The transition of coronaviruses to human hosts has resulted in acute respiratory diseases in humans. The Severe Acute Respiratory Syndrome Coronavirus (SARS-CoV-1), Middle East Respiratory Syndrome Coronavirus (MERS-CoV) and the Severe Acute Respiratory Syndrome Coronavirus 2 (SARS-CoV-2; cause of Coronavirus Disease 2019 or COVID-19) have been associated with extensive outbreaks in 2002–2003 (SARS), clusters of disease from 2012 to 2020 (MERS) and an ongoing 2019–2020 COVID-19 pandemic (Menachery et al., 2017; Jiang et al., 2020; WHO, 2020; Xu et al., 2020). The infectivity doses for human disease by SARS-CoV-2 and other coronaviruses have yet to be defined (Peeri et al., 2020; Sahin et al., 2020). A dose-response model developed for SARS-CoV-1 indicated that 50% of the exposed individuals would develop illness when exposed to 280 plaque forming units of the virus (Watanabe et al., 2010). Given the gaps in our knowledge, the magnitude of the risk due to virally contaminated surfaces is uncertain and should be examined further.

**Figure 1:**
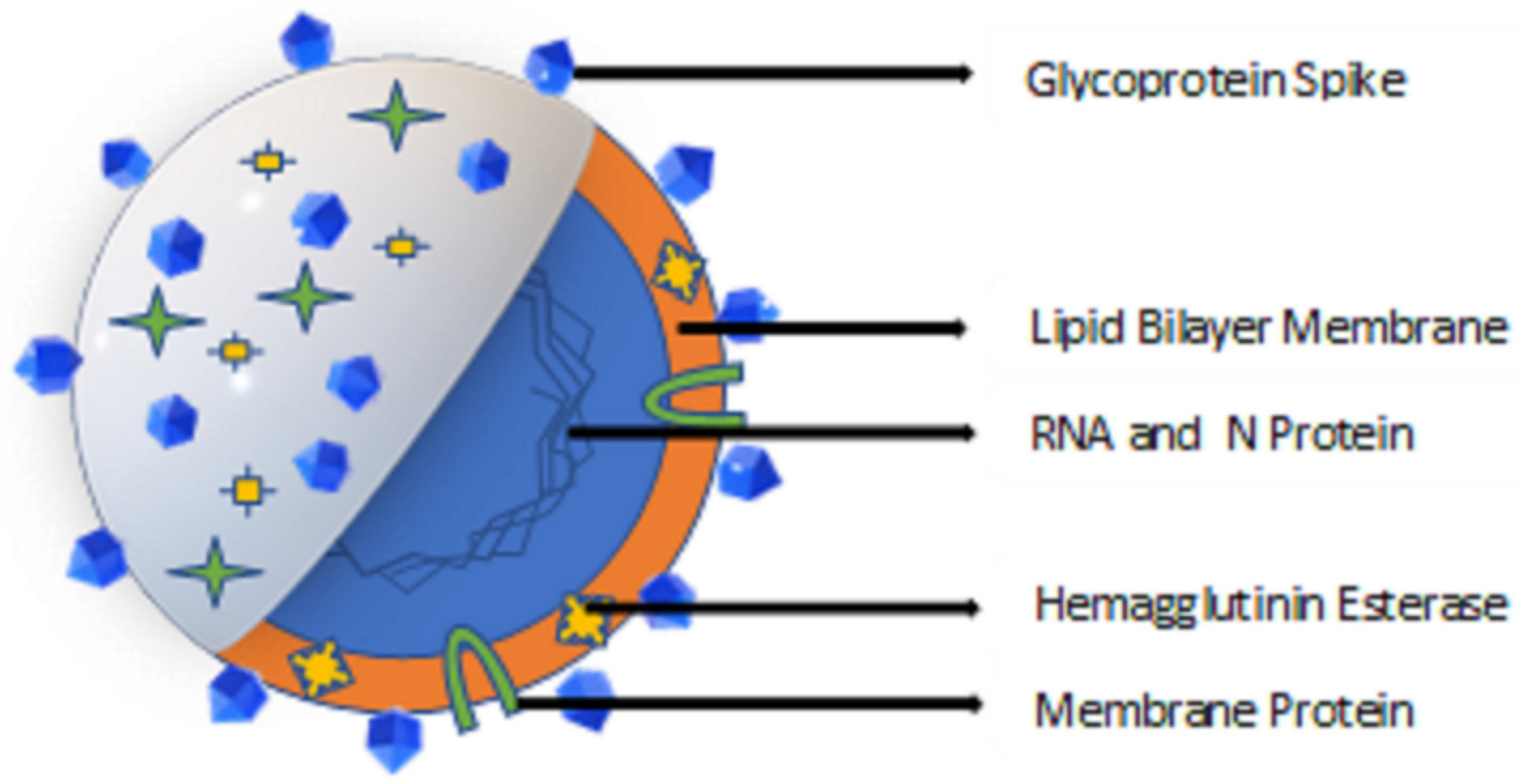
Overview of Coronavirus structure.

Coronaviruses are positive-stranded RNA viruses with an envelope containing glycoprotein spikes. The 26–32 kb genomes of coronaviruses are some of the largest among RNA viruses. While the targets of antiviral drugs against the coronavirus that causes COVID-19 could include its unique glycosylated spike and the M^pro^ viral protease (Jin et al., 2020) (Figure 1), curtailing the spread of the virus remains the first line of defense and a crucial step to reduce the spread of the disease. Disinfectants and biocides effective against coronaviruses may work by inactivating the enveloped virus due to their affinity for the lipid-containing viral envelope, the capsid, and the genome (Pratelli, 2007). The use of antimicrobials for hand sanitation (Hulkower et al., 2011), fomite disinfection, and as nasal sprays and oral rinses (Eggers et al., 2015b; Graf et al., 2018), may reduce human-to-human transmission of the virus. The ongoing COVID-19 outbreak has resulted in shortages of commercial alcohol-based sanitizers, rubbing alcohol, and personal protective equipment (PPE); therefore, this review is intended to provide information regarding a range of alternative biocides effective in eliminating or reducing the presence of coronaviruses from fomites and other potential sources of cross contamination.

## SARS-CoV-2 Transmission Among Food Workers and Food Processing Facilities

**Figure 2:**
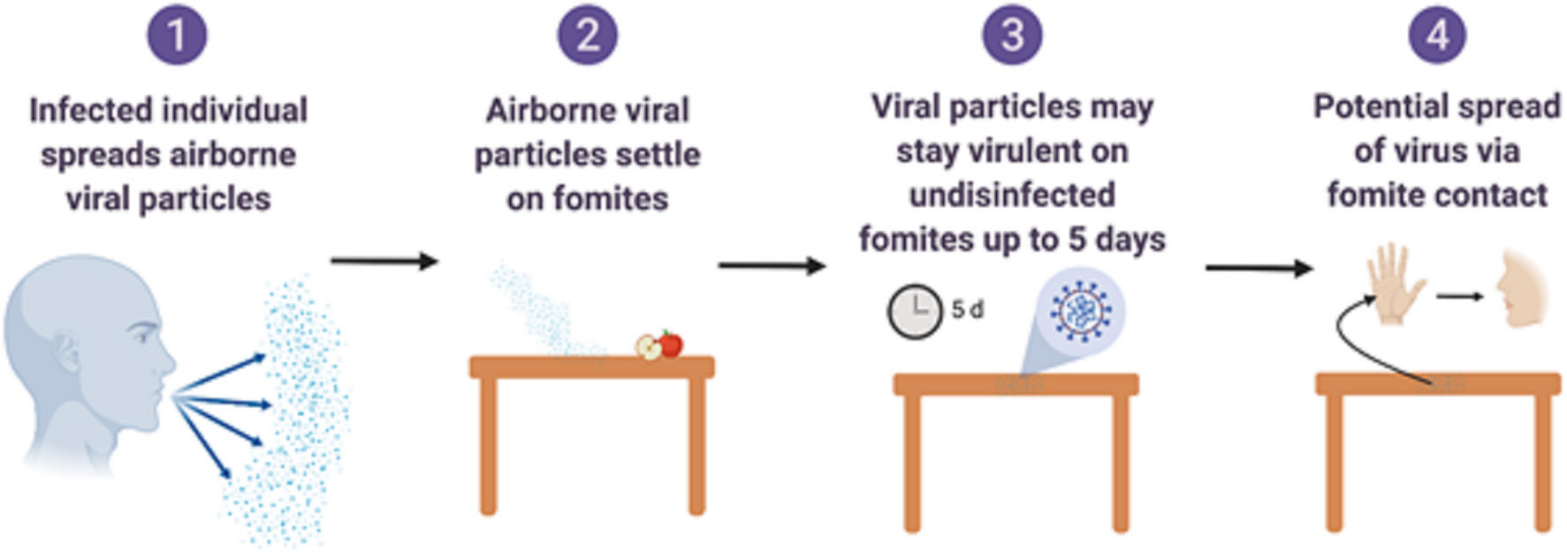
Overview of fomite and surface associated spread of respiratory coronaviruses. Created with BioRender.com.

**Figure 3:**
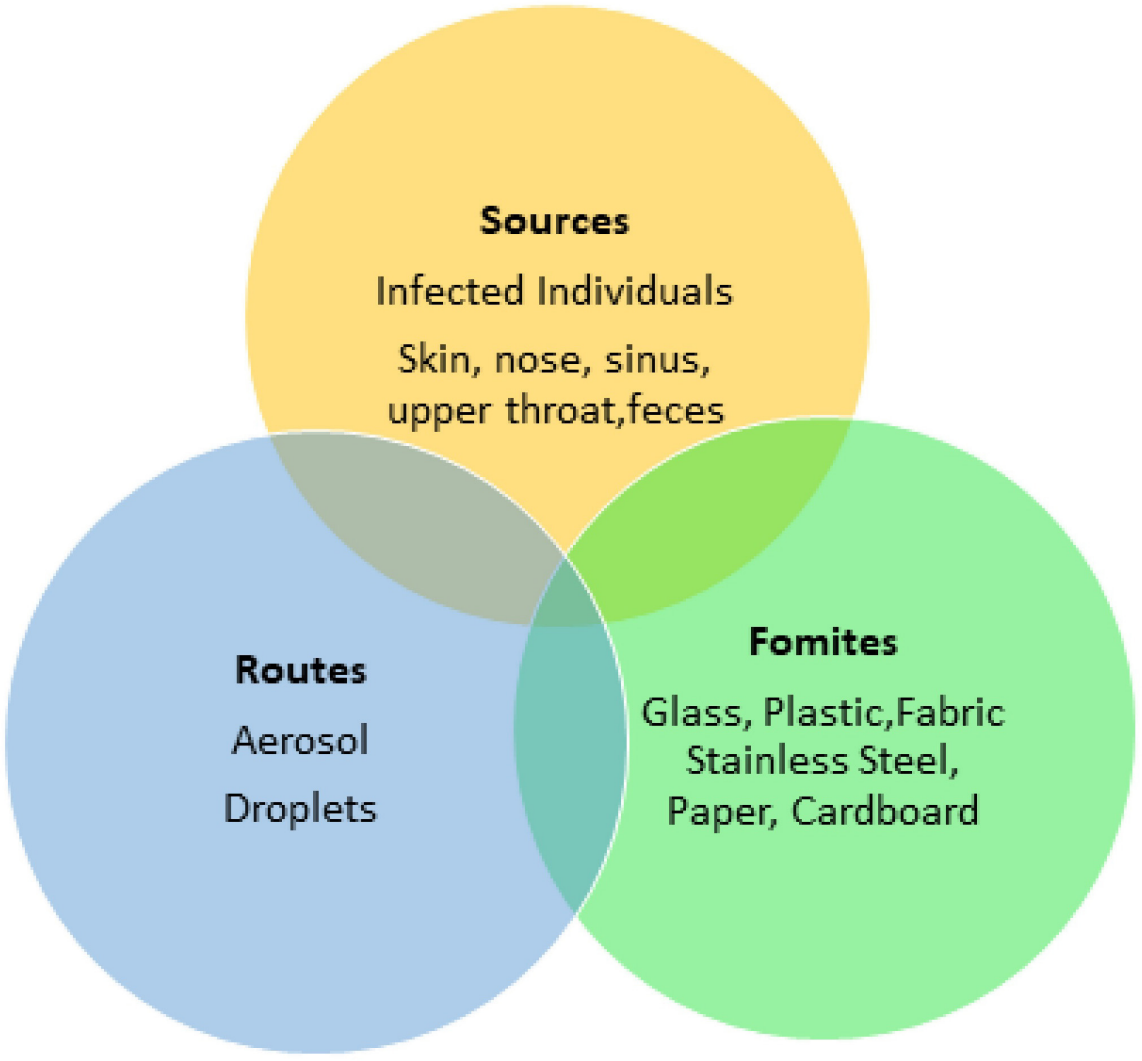
SARS-CoV-2 contagion overview.

Food processing plants and retail facilities often contain a high density of workers working in close proximity. The rapid spread of the SARS-CoV-2 virus indicates that its transmission may be multifactorial (Otter et al., 2016) (Figures 2, 3) such as though aerosols, droplets and fomites. While many food processing facilities have hazard analysis critical control points (HACCP) plans that involve cleaning, sanitation and hand washing programs, several facilities have reported increased spread of SARS-CoV-2 among workers, resulting in shut downs and possible food shortages (Hart et al., 2020).

Transmission of SARS-CoV-2 is different from that of foodborne bacterial pathogens and viruses, which are transmitted via the fecal-oral route. The transmission of SARS-CoV-2 can not be controlled only through hand washing and sanitizer use and requires interventions that prevent aerosol and droplet based transmission of the virus. A recent study of SARS-CoV-2 infected patients indicated that higher viral loads existed in the nose than the throat (Zou et al., 2020). Nasal shedding of virus particles was similar to that the influenza virus (Zou et al., 2020), with both symptomatic and asymptomatic individuals having similar viral loads during the first 10 days of infection, after which individuals with severe illness have a 60 fold increase in viral load (Liu et al., 2020). Control of respiratory transmission of SARS-CoV-2 through aerosols and droplets, as well as transmission by contact with contaminated fomites (Figure 3) requires synergy between conventional and novel techniques, including oral and nasal rinses with approved antimicrobials, as well as hand washing, donning of face masks and social isolation (Bali and Chaudhry, 2020).

SARS-CoV-2, similar to SARS-CoV-1, can remain viable in aerosols for a duration of 3 h. Recent studies on SARS-CoV-2 and previous studies on SARS-CoV-1 and MERS-CoV indicate that coronaviruses can survive on surfaces such as plastics and stainless steel for durations up to or exceeding 5 days (Sizun et al., 2000; Casanova et al., 2010; Van Doremalen et al., 2020). Shorter survival of SARS-CoV-2 was observed on printed and tissue papers, but the virus was recovered from the surfaces of surgical masks after 7 days (Chin et al., 2020). Further recovery of SARS-CoV-2 from wood and fabric for up to 2 days indicates that commonly encountered surfaces can harbor SARS-CoV-2 (Casanova et al., 2010; Otter et al., 2016). Studies using the endemic human coronavirus strain (HCoV) 229E indicate that the coronavirus may maintain infectivity for a week's duration on inert surfaces, while the transmissible gastroenteritis virus (TGEV), another coronavirus, survived for a month at 4°C. Similarly, longer durations of virus recovery (14 days) were observed at 4°C for SARS-CoV-2 (Chin et al., 2020). Factors such as viral load and humidity also influence the survival of coronaviruses. While intervention efforts such as quarantining, distancing of individuals, hand washing, and the disinfection of fomites, including food contact surfaces (WHO, 2020) have been suggested, the potential for mitigation strategies to reduce SARS-CoV-2 viral load, shedding in patients and survival in the environment and on contact surfaces need to be addressed. The objective of this review is to describe antimicrobial agents with virucidal activity against coronaviruses that can be effectively used for sanitation and disinfection of surfaces individually, or in combination to provide effective hurdles to the spread of SARS-CoV-2. Agents for the sanitation and disinfection of carriers, vehicles and fomites in food production, distribution, and retail settings (Sizun et al., 2000; Otter et al., 2016) are the primary focus.

## Fomites as Vehicles and Reservoirs of Coronaviruses

Fomites likely place a role in viral transmission because they can be contaminated with virus-containing secretions, such as aerosols or droplets, expelled through coughing or talking (Figure 2) (Hulkower et al., 2011; Menachery et al., 2017; Kampf et al., 2020). During the 2002–2003 SARS-CoV-1 outbreak, 31 cases in three separate clusters were linked to a single index patient at National Taiwan University Hospital. The third cluster included six healthcare workers with direct SARS patient contact, and six additional infected healthcare workers who had no direct contact with the patient. Contaminated fomites were a suspected route of transmission to the workers with no direct patient contact. Out of 119 environmental samples collected throughout the hospital, nine were confirmed SARS-CoV-1 RNA positive (Chen et al., 2004).

Several other surveys on coronaviruses such as SARS-CoV-1 and MERS-CoV have indicated that fomites, along with airborne routes, contribute to the spread of coronaviruses (Otter et al., 2016; Xiao et al., 2017). Surface swabs of two hospitals treating MERS-CoV patients indicated that 42 out of 68 surfaces were positive for the coronavirus using reverse transcriptase polymerase chain reaction (RT-PCR). The MERS-CoV was cultured from surfaces such as stethoscopes, doorknobs, bed guardrails, and elevators (Kim S.-H. et al., 2016). Swabs of surfaces such as a refrigerator handle, table, and television remote control were positive for SARS-CoV-1 using RT-PCR in SARS units during an outbreak in Toronto (Dowell et al., 2004). Several factors, including surface material, organic load, viral load, temperature, and environmental humidity may influence the survival of viral particles on surfaces (Kim S.-H. et al., 2016; Otter et al., 2016).

A comparative study of the SARS-CoV-1 and SARS-CoV-2 examined viral stability kinetics in aerosols and on surfaces and determined that decay rates for both viruses were similar on many, but not all surfaces (Van Doremalen et al., 2020). Airborne titer reductions for both aerosolized viruses were <1 log_10_ TCID_50_/mL after 3 h. SARS-CoV-1 and SARS-CoV-2 were both detectable on plastic and stainless steel for up to 72 h; SARS-CoV-2 titers decreased from 10^3.7^ to 10^0.6^ TCID_50_/mL after 48 h on stainless steel and after 72 h on plastic. However, SARS-CoV-1 appears to have a significantly shorter half-life on cardboard, as the study found no detectable SARS-CoV-1 after 8 h, while viable SARS-CoV-2 was undetectable after 24 h (Van Doremalen et al., 2020). This suggests that the causative agent of the current COVID-19 pandemic might survive better on environmental surfaces than SARS-CoV-1; however, the authors did caution that considerable statistical dispersion occurred within studies examining cardboard (Van Doremalen et al., 2020). Coronaviruses (HCoV-229E and feline infectious peritonitis virus or FIPV) can remain infectious for long periods in water (>100 days in water at 4 °C and >10 days in water at 23°C) and pasteurized settled sewage (2–4 days), suggesting contaminated water may be a potential vehicle for human exposure if aerosols are generated (Gundy et al., 2009). At 25°C, the time required for a 99% reduction in reagent-grade water was 22 days and 17 days for two SARS surrogates, TGEV and MHV, respectively. In settled sewage that was pasteurized to reduce competing microorganisms and then spiked with coronaviruses, times for a 99% reduction were 9 days for TGEV and 7 days for MHV. At 4°C, there was <1 log_10_ infectivity decrease for both these surrogates after 4 weeks (Casanova et al., 2009). However, in wastewater, domestic sewage, and dechlorinated tap water, inoculated SARS-CoV-1 persisted for 14 days at 4°C but only for 2 days at 20°C.

While several factors affect the survival and infectivity of SARS-CoV-2 such as the type of surface (stainless steel, plastics and cardboard), moisture level and contaminants (protein, saliva and fecal material), the risk of SARS-CoV-2 presence on food contact surfaces and packing materials remains high during a pandemic. Infected individuals albeit asymptomatic could come in contact with food or packaging throughout the food supply chain; hence the use of mitigation strategies should also be considered from food production facility to consumer handling of the food product.

## Importance of Judicious Biocide Selection

Though the current SARS-CoV-2 literature indicates that it is a respiratory virus and not a food safety concern, coronaviruses and other respiratory viruses have been known to survive on produce such as lettuce for several days (Yépiz-Gómez et al., 2013). SARS-CoV-2 has been isolated from feces, indicating that shedding through the gastrointestinal system occurs (Yeo et al., 2020); therefore, it is hypothesized that SARS-CoV-2 may also have an affinity for cells in the intestine and colon (Gu et al., 2020).

Workers in the food, retail, service, and health industries normally come in close contact with a large number of individuals during the work day. Recent shut downs of meat processing facilities that have established sanitation programs due to spread of SARS-CoV-2 among workers indicates the importance for new control strategies to spread the transmission of the virus (Hart et al., 2020). Hence efforts to minimize the risk of virus contamination of common contact surfaces and survival of the virus in droplets and aerosols in food manufacturing, production, and retail centers should be considered. These efforts include the use of antimicrobial agents such as sanitizers and disinfectants on hands and fomites (Otter et al., 2016; Eggers, 2019). The improper selection and inadequate use of sanitizers and disinfectants plays a significant role in the cross transfer and spread of pathogens (Hirose et al., 2019) resulting in additional public health concerns. Sanitizer choice and coronavirus susceptibility to current cleaning and sanitation practices within facilities is an important consideration. For instance, coronaviruses such as the mouse hepatitis virus (MHV) and TGEV are less susceptible to 1:100 hypochlorite than they are to 70% ethanol (Hulkower et al., 2011). Over-dilution of sanitizers and insufficient product contact time are critical factors that should be taken into account when targeting the elimination of coronaviruses from fomites (Boyce, 2016).

The presence of SARS-CoV-2 in human feces (Xiao et al., 2020) highlights the importance of incorportating cleaning and disinfection regimens in toilets and restrooms as well as developing protocols to prevent aerosolization of virus particles during flushing. The use of quartenary ammonium (alkyl dimethyl benzyl ammonium chloride) and peracetic acid was effective in coliphage MS2 redution on surfaces after flushing (Sassi et al., 2018). The use of biocides effective against SARS-CoV-2 in toilet bowls apart from cleaning could be considered in light of the information presented in this review.

## Efficacy of Biocides Against Coronaviruses

### Estimation of Virucidal Activity

Virucidal activity can be determined by suspension tests as well as carrier tests that mimic surfaces and evaluate the performance of biocides in the presence of organic loads through the addition of serum. Viral reduction is determined by infectivity assays where treated viruses are compared to untreated controls for the reduction in viral cytopathic effects (CPE) on tissue culture monolayers. Surviving fractions determined through Log_10_ reductions are enumerated either by viral plaque assays, a most probable number (MPN) assay, or by determining the 50% titration endpoint for infectivity (known as tissue culture infectious dose 50% or TCID_50_ assay). Plaque forming units (PFU) are proportional to TCID_50_ titer by a factor of 0.56 (Wulff et al., 2012). RT-PCR for the estimation of viral nucleic acid using threshold cycle (Ct value) has also been used to determine viral load. Reduction factors are calculated using the difference in the quotient of the infection titer before and after exposure to the antimicrobial agent (Rabenau et al., 2005b) According to the Environmental Protection Agency (EPA), an acceptable disinfectant claim requires a 4 log_10_ reduction in the human norovirus surrogate, feline calicivirus (FCV) (EPA, 2017). European countries recommend the use of other viral surrogates such as murine norovirus (MNV) or adenovirus type 5 (AdV-5) for testing (Rabenau et al., 2014). Criticisms for suspension tests include that they do not mimic “real world” conditions, which should be taken into consideration while determining virucide selection for disinfection of surfaces soiled with organic matter and other substances that could reduce efficacy. Results regarding the virucidal activity of disinfectants using non-enveloped surrogates translate well to the more susceptible enveloped viruses, such as coronaviruses. Several factors such as target strain, testing of biocide against virus in suspension vs. drying and use of protein supplementation play important roles in influencing viral particle stability and loss of infectivity during the determination of virucidal activity (Rabenau et al., 2005a).

### Alcohol and Alcohol Based Sanitizers

Alcohol based sanitizers can be used on skin, for the disinfection of fomites and on certain food contact surfaces (Table 1). Alcohol acts on viral envelopes to denature proteins and is not significantly impaired by organic matter contamination (Springthorpe et al., 1986). Ethyl alcohol (ethanol), isopropyl alcohol (isopropanol or rubbing alcohol), and 2,4 dichlorobenzyl alcohol are classes of alcohol that have been shown to possess antimicrobial properties, although their concentrations and ranges of activity differ (Lambert, 2004).

**Table 1:** Overview of applications for biocides that are effective against coronaviruses.

**Active ingredient(s)**	**Applicable surface (skin, fomites, air)**	**Food contact (Yes or No)**	**Concentration or Level^a^**				**References**
			**Food contact surfaces (Zone 1^b^)**	**Non-food Contact (Zones 2^c^ & 3^d^)**	**Skin**	**Aerosol**	
Ethyl alcohol (ethanol)	Skin, fomites	Yes	70% (v/v)	70 - 95% (v/v)	80% (v/v)	N/A	Rabenau et al., 2005b; Kampf et al., 2020
Isopropyl alcohol (isopropanol)	Skin, fomites	Yes	70% (v/v)	60–90% (v/v)	75% (v/v)	N/A	Kratzel et al., 2020
Povidone iodine	Skin, fomites	No	N/A		5–10% (v/v)	N/A	Gaulin et al., 2011
Quaternary ammonium	Fomites	Yes	<200 ppm	200 ppm	N/A	N/A	Gaulin et al., 2011
Hydrogen peroxide	Skin, fomites	Yes	35% (v/v)		0.125% (v/v)	N/A	Gaulin et al., 2011
Sodium hypochlorite	Fomites	Yes	100–200 ppm	>200 ppm	N/A	N/A	Gaulin et al., 2011
Peroxyacetic acid (PAA)	Fomites	Yes	5–500 ppm	500 ppm	N/A	N/A	Gaulin et al., 2011
Chlorine dioxide	Fomites, air	Yes	3 ppm		N/A	0.03 ppm	Miura and Shibata, 2010
Ozone	Fomites, air	Yes	2 ppm		N/A	<0.05 ppm	Hudson et al., 2007, 2009
Ultraviolet (UV) light	Fomites, air	Yes	200–280 nm		N/A	200–280 nm	Kariwa et al., 2006; Walker and Ko, 2007

a *Recommended by US Environmental Protection Agency (Values on table represent general ranges according to the EPA. Always follow EPA label instructions for specific antimicrobial pesticide formulations)*.

b *Zone 1: Food-Contact Surfaces*.

c *Zone 2: Non-food-contact surfaces in close proximity to food and food contact surfaces*.

d *Zone 3: More remote non-food-contact surfaces that are in or near the processing areas and could lead to contamination of zones 1 and 2*.

*N/A, Not applicable*.

Hand sanitizers and rubs containing alcohol (75–85% v/v) effectively reduce the infectivity of coronaviruses in *in vitro* tests. Against SARS-CoV-2, both the World Health Organization sanitizer formulation 1 [85% ethanol (v/v), 0.725% glycerol (v/v) and 0.125% hydrogen peroxide (v/v)] and formulation 2 [75% isopropanol (w/w), 0.725% glycerol (v/v) and 0.125% hydrogen peroxide (v/v)] resulted in complete inactivation from an initial viral titer of 8 log_10_ (TCID_50_/ml). Tests of both ethanol and isopropanol within the same study (along with 0.125% hydrogen peroxide) against SARS-CoV-2 were effective in inactivating the virus within 30 s, even when used at a concentration of 30% (Kratzel et al., 2020), Products based on 80, 85, and 95% ethanol without dilution inactivated SARS-CoV-1 to below the limit of detection (RF ≥ 4) within 30 s of exposure (Rabenau et al., 2005b). High concentrations of ethanol (95% and 85%) based hand sanitizers have also been useful in reducing SARS-CoV-1 by 5.5 log_10_ (TCID_50_/ml) within an exposure time of 30 s (Rabenau et al., 2005b).

Ethanol at a concentration of 70% resulted in a 3 log_10_ reduction of coronaviruses (TGEV and MHV) after an exposure duration of a minute. Hand sanitizers with 62% ethanol resulted in a reduction factor of 4 log_10_ of TGEV and a 2.7 log_10_ reduction of MHV (Hulkower et al., 2011). Alcohol-based formulations containing 3.2% povidone-iodine and 78% alcohol reported 99.99% (4 log_10_ reduction) inactivation of the modified vaccinia virus Ankara (MVA), a reference virus for virucidal hand disinfectants, under clean and dirty conditions after a 15 s contact time (Eggers et al., 2015a) indicating that these sanitizers might also be effective against other enveloped viruses such as coronaviruses. When evaluated on inanimate surfaces like metal, glass, or plastic, 78–95% ethanol inactivated the coronaviruses SARS-CoV-1, MERS-CoV, and MHV to reduction factor ≥4 in 30 s (Kampf et al., 2020). The use of amyl metacresol (0.6 mg) and dichlorobenzyl alcohol (1.2 mg) at pH of 2.3 in throat lozenges resulted in negligible antiviral activity against human coronavirus OC43 (hCoV OC43) (Morokutti-Kurz et al., 2017) in *in vitro* tests.

### Povidone Iodine and Povidone Iodone Based Products

Povidone Iodone (PVP-I) has been used for skin, nasal, and oral cavity disinfection (Table 1). PVP-I is an iodophore with broad spectrum antimicrobial activity against bacteria, fungi, and viruses. PVP-I forms I_2_ and hypoiodous acid (HOI), which oxidizes nucleic acids and membranes (Lachapelle et al., 2013). PVP-I is used for the disinfection of skin when formulated into scrubs or hand washes and for oral cavities through oral sprays and mouth rinses (Nagatake et al., 2002; Kariwa et al., 2004; Durani and Leaper, 2008). Nasal spray of PVP-I has been used for the post-operative control of *Staphylococcus aureus* infections and could potentially be used to reduce nasal harborage and dispersal of SARS-CoV-2 (Phillips et al., 2014). The exposure of SARS-CoV-2 (7.8 of log_10_ (TCID_50_/ml) to 7.5% of PVP-I resulted in the virus titer dropping below levels of detection after 5 min (Chin et al., 2020). The use of PVP-I at a concentration of 7.5% (surgical scrub), 4% (hand wash), and 1% + 8.3% alcohol (mouth rinse) against MERS-CoV resulted in a 99.99% reduction in virus populations after 15 s in both clean and soiled conditions (Bovine serum albumin and erythrocytes). Virucidal activity of PVP-I was observed against MERS-CoV even after a 1:10 dilution, though a higher duration of exposure (30 s) was required for the oral rinse that contained 1% PVP-I + 8.3% alcohol (Eggers et al., 2015b).

Antiviral activity of PVP-I containing products (0.23–1%) was observed against SARS-CoV-1. Exposure of SARS-CoV-1 to PVP-I containing products reduced a viral load of 1.17 × 10^6^ TCID_50_/ml to below levels of detection within a duration of 2 min (Kariwa et al., 2004). PVP-I was also effective when used against human rotavirus, a non-enveloped virus that causes diarrhea, on disk of stainless steel and plastics, indicating its effectiveness as a surface sanitizer (Lloyd-Evans et al., 1986). The efficacy of PVP-I against test bacterial pathogens (skin contaminant surrogates) did not decrease when tested on an inert surface (Durani and Leaper, 2008), indicating that PVP-I could be used for hand washing and disinfection of skin, surfaces and the oral tract, and as a substitute or replacement for alcohol-based products (Durani and Leaper, 2008). While PVP-I can stain surfaces, it is water soluble and stains can be washed away or removed with a damp cloth. PVP-I' virucidal efficacy against coronaviruses at concentrations as low as 0.23%, rapid efficacy at 15 s, and residual efficacy in combination with isopropyl alcohol or ethanol make it an excellent choice for disinfecting skin, oral cavities, and fomite surfaces (Eggers et al., 2015b; Kampf et al., 2020). The combination of PVP-I with alcohol as a disinfectant could reduce the amount of alcohol required and could serve as a useful substitute or supplement to alcohol use.

### Quaternary Ammonium Compounds and Quaternary Ammonium Compound Based Disinfectants

Quaternary Ammonium Compounds (QACs) are popular sanitizers that can be used on certain food contact surfaces as well as fomites (Table 1). QACs are cationic detergents with membrane active properties, and their antimicrobial activity is due in part to their ability to disrupt the lipid membrane of a microorganism (Rabenau et al., 2005b; Pratelli, 2007; Kumar et al., 2017). The effectiveness of QACs is very formulation specific and this affects the range of organisms to which they are effective and the time needed to be effective against a specific organism (Gerba, 2015) The exposure of SARS-CoV-2 (7.8 of log_10_ TCID_50_/ml) to 0.10% (100 ppm) of benazlkonium chloride resulted in viral titer reduction below levels of detection after 5 min (Chin et al.). An analysis of the efficacy of household disinfectants against murine hepatitis virus (MHV), a surrogate for SARS-CoV-1, indicated that a formulation of 0.10% (100 ppm) quarternary compound with 79% ethanol resulted in a 3 log_10_ (TCID_50_/ml) reduction after a 30 s exposure time (Dellanno et al., 2009). The use of 1% (1,000 ppm) benzalkonium-chloride (a QAC) and 1% (1,000 ppm) chlorhexadine digluconate (a polybiguanide) against SARS-CoV resulted in a loss of culturability of the virus, though detection of viral RNA through PCR occurred 30 min after exposure (Ansaldi et al., 2004). The formulations and test conditions used by Kampf et al. (2020) indicated a low efficacy against MERS-CoV. The use of ethanol along with QACs usually has been associated with effective antimicrobial activity against coronaviruses (Sattar, 2004).

### Hydrogen Peroxide

Hydrogen peroxide is commonly used to disinfect food contact surfaces, as a fumigant and as a sanitizer (Table 1) (Kumar et al., 2017). Studies have shown that hydrogen peroxide is effective against SARS-CoV and its surrogates. Exposure of a coronavirus surrogate (TGEV) dried on stainless steel to hydrogen peroxide vapor (20 μl) for 2–3 h resulted in approximately a 5 log_10_ (TCID_50_/ml) reduction (Goyal et al., 2014). A limitation to this study was that the hydrogen peroxide vapor was examined on clean surfaces; therefore, further studies examining the impact of organic material and soil are necessary to determine its efficacy in a range of environments and situations. Another study using a commercial product (ACCEL TB) containing liquid hydrogen peroxide with surfactants was effective (>4 log_10_ TCID_50_/ml reduction) at a concentration of 0.5% with an incubation time of 1 min against HCoV-229E (Omidbakhsh and Sattar, 2006). However, limited information exists regarding the virucidal activity of hydrogen peroxide on other types of surfaces.

### Sodium Hypochlorite

Sodium hypochlorite (NaOCl; chlorine bleach) has been used as disinfectant for the past century in water and on food contact surfaces (Table 1; Kumar et al., 2017). Hypochlorous acid (HOCl) and the hypochlorite ion contribute the majority of the disinfectant activity associated with bleach-containing products, with the former compound contributing the most biocidal activity (Kott et al., 1975; Rutala and Weber, 1997). However, while chlorine-derived compounds do exhibit significant efficacy against coronaviruses on non-porous surfaces, organic matter and porous materials diminish virucidal activity because of the quenching of free chlorine (Geller et al., 2012). Common practice in the food industry is to adjust alkaline chlorine formulations to ca. pH 7 using a food grade acid when it is used at higher concentrations to increase dissociation into the more potent antimicrobial compound HOCl. However, pH adjustment is less common in healthcare and household environments and does not occur in literature examining the virucidal activity of bleach in these settings (Kott et al., 1975).

The exposure of SARS-CoV-2 (7.8 of log_10_ (TCID50/ml) to 1:49 (~150 ppm) and 1:99 (~75 ppm) household bleach resulted in the virus titer being reduced below levels of detection after 5 min (Chin et al., 2020). To elucidate the target of antiviral activity, bovine coronavirus was exposed to 100,000 ppm NaOCl (pH 11.5) for 1 min. Real-time reverse transcriptase PCR (rRT-PCR) and Western Blot indicated that total RNA and nucleoprotein degradation occurred in that time period. When the concentration was reduced to 10,000 ppm NaOCl, a 10 min treatment was required to achieve complete nucleoprotein degradation, although there was <1 log_10_ reduction in total RNA units observed (Bieker, 2006). HCoV-229E challenged with 5,000 ppm NaOCl for 10 min on an inanimate surface underwent a ca. 3 log10 (TCID50/ml) reduction, which failed to meet EPA standards for virucidal activity for a disinfectant claim (Tyan et al., 2018). However, when treatment levels were increased to 2,100 ppm NaOCl) on stainless steel coupons, a ≥4.5 log_10_ TCID50/mL reduction was achieved after 30 s against the SARS-CoV-1 surrogate, MHV (Dellanno et al., 2009). Much lower concentrations were required in seeded hospital wastewater; SARS-CoV-1 was inactivated by exposure to 10 ppm NaOCl (0.4 ppm free chlorine) after 10 min exposure, while inactivation occurred within 1 min in 20 ppm NaOCl (0.5 ppm free chlorine; Kott et al., 1975; Rutala and Weber, 1997; Kapil et al., 2004; Wang et al., 2005; Dellanno et al., 2009; Geller et al., 2012; Kumar et al., 2017; Chin et al., 2020).

### Peroxyacetic Acid and Acetic Acid

Uses of peroxyacetic acid (PAA) include the sanitation of food contact surfaces and for post-harvest produce washing. The antimicrobial action of PAA involves the production of reactive oxygen species (ROS) (Vandekinderen et al., 2009). ROS oxidize sulfhydryl and disulfide bonds, which in bacteria leads to increased cell wall permeability, impacted enzymatic transport systems, and disrupted cell membranes (Vandekinderen et al., 2009). While PAA has shown effectiveness on bacterial pathogens on food and food contact surfaces, it has varied impact on foodborne viruses, notably human norovirus (NoV) and hepatitis A virus (HAV), both non-enveloped viruses which tend to be more resistant to antimicrobials than enveloped viruses (Watanabe et al., 1989; Barker et al., 2001). A PAA-based biocide (100 ppm PAA) used to wash lettuce had no significant disinfection effect on viral titers of HAV and murine norovirus (MNV) (Fraisse et al., 2011). Higher concentrations of PAA (>100 ppm) may be necessary to reduce non-enveloped viruses on surfaces, foods, and fomites, and research regarding the effectiveness of PAA on coronaviruses is limited. A 0.035% (35 ppm) solution of PAA inhibited SARS-CoV-1 replication in cell culture with <2 min of contact time (Ansaldi et al., 2004), while the same concentration did not affect the viral genome after 30 min of exposure (Ansaldi et al., 2004). Another study suggested that SARS-CoV-1 can be inactivated with 500 to 1,000 ppm of PAA (Wang et al., 2005). The EPA has listed several PAA-based sanitizers and disinfectants that can be used against SARS-CoV-2, in addition to other viruses (https://www.epa.gov/pesticide-registration/list-n-disinfectants-use-against-sars-cov-2; accessed March 24, 2020). Wine vinegar (6% acetic acid) was effective in inactivating SARS-CoV-1 by a reduction factor of 3 log_10_ within an exposure duration of 30s (Rabenau et al., 2005a) providing both processers and consumers with an option for food contact surface disinfection.

### Chlorine Dioxide

Chlorine dioxide (ClO_2_) is a gas at room temperature and is easily dissolved in water, although concentrations in water diminish rapidly (Gates et al., 2009). It is an effective disinfectant in both gas and liquid states, making it a versatile biocidal agent (Gates et al., 2009; Morino et al., 2011). A ClO_2_ solution at concentrations yielding 2.19 ppm free chlorine in wastewater has been reported to inactivate SARS-CoV-1 (Wang et al., 2005; Miura and Shibata, 2010), which makes it a less efficacious disinfectant against the virus than chlorine, which was effective at 0.5 ppm free chlorine. To achieve complete inactivation of the virus in wastewater, ClO_2_ at 20 ppm required a 5 min contact time. However, a 10 ppm solution only achieved a 55.3–68.4% inactivation of the virus (Wang et al., 2005).

ClO_2_ is an active virucidal agent in its gaseous state. When placed in an environment with chlorine dioxide at concentrations of 0.05 ppm, Influenza A virus (an enveloped virus) on wet glass slides was reduced from > 6 log_10_ TCID_50_ to below the limit of detection (<0.5 log_10_ TCID_50_) within 3 h, while the control (air) titers remained unchanged after 5 h exposure (Morino et al., 2011). Complete inactivation of SARS-CoV surrogate MHV strain A59 after 12 h exposure to 0.16 ppmv/min ClO_2_ gas has been reported, with titers reduced 3.5 times after 6 h exposure (Kim J. et al., 2016). ClO_2_ can also be safely used in low concentrations around animals and people to control airborne viruses. Mice housed in an environment with 0.032 ppm ClO_2_ were exposed to aerosolized influenza virus A and compared to mice housed in fresh air with no ClO_2_. After 3 days, pulmonary titers in the control group were 6.7 TCID_50_, significantly higher than the 2.6 TCID_50_ observed within the mice exposed to ClO_2_ (Miura and Shibata, 2010). Gaseous oxidizers should be used according the federal regulations and should be monitored to prevent inadvertent exposure to personnel (CDC, 1978).

### Ozone

Ozone is a naturally occurring configuration of three oxygen atoms and has a half-life of about 1 h at room temperature; degradation results in spontaneous oxygen gas formation (Kumar et al., 2017). A powerful oxidant, ozone has unique biological properties and can be used as a gas at recommended levels with monitoring and can also be dispersed in water. Viral susceptibility to ozone varies. Enveloped viruses such as coronaviruses might be more sensitive than non-enveloped viruses due to the interaction of ozone with the lipid layer envelopes (Kumar et al., 2016). Zhang et al. (2004) reported that a high concentration of 27.73 ppm ozone inactivated SARS-CoV-1 in 4 min. The medium (17.82 ppm) and low (4.86 ppm) concentrations could also inactivate SARS-Cov-1 with different speeds and efficacy (Zhang et al., 2004). In another study, maximum anti-viral efficacy of ozone required a short period of high humidity (>90% relative humidity) after the attainment of peak ozone gas concentration (20–25 ppm) (Hudson et al., 2007). Mouse coronavirus (MCoV) on different surfaces (glass, plastic, and stainless steel) and in the presence of biological fluids was inactivated by ozone by at least 3 log_10_ in the laboratory and in simulated field trials (Hudson et al., 2007, 2009). Ozone can be harmful to personnel when inhaled and should be used according to federal regulations (CDC, 2019). Precautions should be taken to monitor ozone levels in air to avoid inadvertent exposure to personnel (CDC, 2019).

### Ultraviolet Light

Ultraviolet (UV) light has three classifications (UVA, UVB, and UVC) based on wavelength and is known to cause pyrimidine dimers and breakage in nucleic acids (Tseng and Li, 2005). This dimerization disrupts transcriptional and translational processes, affecting cellular function and can thus also interfere with viral replication. UV light treatment can be employed to target three transmission forms of viral particles: (1) in droplets, (2) aerosolized, and (3) on fomites; however, the inactivation of coronaviruses via UV light can be challenging as inactivation rates vary based on wavelength and the length of the RNA transcript (Stern and Sefton, 1982). Generally, inactivation rates increase with the length of the RNA transcript (Stern and Sefton, 1982). Also, UV target sizes for viral messenger RNA (mRNA) are typically directly related to that of the genomic-size RNA (Yokomori et al., 1992).

UVC light (254 nm) with an intensity of 4,016 μW/cm^2^ inactivated SARS-CoV-1 in a liquid medium at a 3 cm distance for 15 min, while UVA light had no effect on viability (Darnell et al., 2004). UV light, in combination with riboflavin, a B vitamin, reduced MERS-CoV titer below the limit of detection of 2.18 log_10_ PFU/mL from an initial concentration of 7.5 log_10_ PFU/mL (Keil et al., 2016). Other studies have examined the effectiveness of UV light on aerosolized viral particles. SARS-CoV-1 in an aerosolized form treated with UV light illustrated a greater susceptibility (Z-value ratio of air to liquid of 85.7) compared to that of the virus in liquid media (Walker and Ko, 2007). While UV light (134 μW/cm^2^) for a duration of 15 min was effective in significantly reducing the infectivity of SARS-CoV-1 from 7.57 to 2.25 log_10_ TCID_50_/mL, the treatment did not completely eliminate the virus (Kariwa et al., 2006). UV light should be used according to federal regulations and during hours when operations have ceased to prevent inadvertent exposure to personnel (21 CFR 880.6600) (FDA, 2019).

### Metals

Very few studies have examined the effectiveness of metals against viruses. In a study by Bright et al. (2009), zeolite powders amended with silver and/or silver/copper ions resulted in reductions of 1.08 log_10_ TCID_50_/ml (3.5% Ag, 6.5% Cu), 0.43 log_10_ TCID_50_/ml (20% Ag) and 0.50 log_10_ TCID_50_/ml (0.6% Ag, 14% Zn, 80% ZnO) of HCoV-229E after 1 h in a saline suspension (Bright et al., 2009). Silver/copper zeolites were the most effective, with an observed 2.06 log_10_ TCID_50_ reduction after 4 h and a 5.13 log_10_ TCID_50_ reduction within 24 h. A 3.18 log_10_ reduction was observed for FIPV (feline coronavirus) after 4 h (Bright et al., 2009). The long duration required for inactivation of coronaviruses by metals such as silver and copper indicate that they might be ineffective in food production operations when used individually as rapid disinfection is required.

Silver has been shown to have antiviral activity against numerous viruses including the enveloped HIV, HSV-1, herpes vesicular stomatitis virus (HSTV), and vaccinia virus, and the non-enveloped papovaviruses and adenovirus (AdV) (Silvestry-Rodriguez et al., 2007). The use of silver as a coating on food contact surfaces and processing equipment could be considered for further testing and validation as silver ions have also been demonstrated to inactivate the non-enveloped poliovirus (PV) and coliphages (Yahya et al., 1992) and synergistic antiviral activity in the presence of oxidizing agents. Similarly, silver has been shown to have synergistic antimicrobial activity against MS-2 bacteriophage when used in conjunction with UV light (Butkus et al., 2004).

### Plant-Based Antimicrobials

Several plant-based compounds, though not biocides, could be effective in reducing the infectivity of SARS-CoV-2 by inhibiting or blocking viral attachment to host cells. Phytocompounds, betulinic acid and savinin (Wen et al., 2007) and essential oils from *Laurus nobilis* (from berries), *Thuja orientalis* (from fruit), and *Juniperus oxycedrus* ssp*. oxycedrus* (from berries) (Loizzo et al., 2008) have been shown to be effective against SARS-CoV-1.

Iota-carrageenan, a generally regarded as safe (GRAS) polymer derived from red seaweed (Rhodophyceae) is a commonly used food thickener that has demonstrated inhibitory activity against coronaviruses and other respiratory viruses (Graf et al., 2018). Iota-carrageenan forms a protective barrier on mucosa when used as a nasal spray, preventing the attachment of the virus to cell surface (Grassauer et al., 2008). Against human coronavirus OC43 (HCoV OC43), iota-carrageenan had an MIC of 0.024 μg/mL (Graf et al., 2018). Iota-carrageenan has demonstrated inhibitory activity against respiratory viruses such as Influenza A H1N1 (Wang et al., 2011) and reduced the viral load in nasal secretions of children displaying acute symptoms of common cold (Fazekas et al., 2012). Common colds in humans can be caused by viruses such as human rhinovirus (hRV), human coronavirus (hCoV), parainfluenza (PIV), influenza (infA and infB), respiratory syncytial virus (RSV), adenovirus(ADV), enterovirus (EV), and metapneumovirus (MPV) (Koenighofer et al., 2014). Exploration of the use of iota-carrageenan (0.12%) nasal spray to prevent common colds caused by these viruses indicated that patients using iota-carrageenan nasal sprays had significantly reduced durations of symptoms, relapses, and viral titers with highest efficacy against hCoV (Koenighofer et al., 2014). Relapses among patients treated with iota-carrageenan nasal sprays were observed less frequently in groups infected with hrv and hCoV (Koenighofer et al., 2014). The use of iota-carrageenan nasal sprays could be used as a method to prevent infection transmission among workers in food processing facilities. Iota-carrageenan is GRAS certified (21 CFR 172.620) and is approved for use in foods, cosmetics and pharmaceuticals (Hebar et al., 2015).

There are numerous groups of plant compounds/components that have been shown to have antimicrobial activity including saponins, thiosulfinates, glucosinolates, terpenoids, and polyphenols. Many of these have been shown to have efficacy against various enveloped viruses such as herpes simplex viruses types 1 and 2 (HSV-1 and HSV-2), bovine herpesviruses (BHV), bovine viral diarrhea virus (BVDV), human immunodeficiency virus (HIV), dengue virus (DENV), junin virus (JUNV), yellow fever virus (YFV), human respiratory syncytial virus (HRSV), influenza A virus (INFV-A; H1N1, H3N2, H5N1, and H9N2 strains), Newcastle disease virus (NDV), viral hemorrhagic septicemia virus (VHSV), human cytomegalovirus (HCMV), and measles virus (MeV) indicating possible efficacy against coronaviruses (reviewed by Goyal and Cannon, 2006; Bright and Gilling, 2016).

## Conclusion

The high infectivity of the COVID-19 coronavirus, SARS-CoV-2, has caused rapid person to person transmission resulting in a pandemic that has posed multifarious challenges to the food industry. Though not transmitted through food, infections caused by SARS-CoV-2 have resulted in the closing of food processing plants due to infections among essential workers. Furthermore food contact surfaces and food packaging materials could serve as fomites for SARS-CoV-2, highlighting the importance of biocide use to mitigate the spread of the virus.

Currently used methods to reduce the transmission of the virus involve the use of masks, social distancing as well as the use of USEPA approved disinfecting and sanitizing agents. These practices have not been fully successful in preventing transmission of SARS-CoV-2 in several food processing facilities. The information presented in this review indicates that SARS-CoV-2 can be transmitted through the air, feces, soiled surfaces and could occur on surfaces that are frequently touched. Our review indicates that ethanol at high concentrations (>70%), povidone iodine, hypochlorite and QACs when combined with alcohol are efficacious against SARS-CoV-2 for surface disinfection. hydrogen peroxide vapor, chlorine dioxide, ozone and UV could be applied to reduce viral load present in aerosols with appropriate precautions to prevent exposure of personnel to these antimicrobials.

While hand washing and the use of sanitizers is a commonly implemented practice in food production plants, the dispersal of the SARS-CoV-2 virus from often asymptomatic individuals carrying high viral loads in their nasal epithelium requires the exploration of new practices such as the use of nasal sprays to minimize person to person transmission of the virus. The review presents information on antimicrobials and plant-based compounds that could be explored to curtail transmission of SARS-CoV-2. Plant derived iota carrageenan could prevent viral attachment to cells and reduce viral loads in the nasal epithelium. Povidone iodine has also been used in nasal sprays and might serve as an additional preventative measure to control the person-person transmission of SARS-CoV-2.

The use of a multiple hurdles to control the spread of pathogenic microorganisms is a common practice in the food industry and hence the implementation of several mitigation strategies can be adapted by the food industry. Biocides effective against SARS-CoV-2 on moist/soiled surfaces, air and skin is a requirement of high priority for transmission control. Food processing facilities should practice the judicious and optimal use of biocides to avert the development of antimicrobial resistance in non-target bacterial pathogens during the SARS-CoV-2 pandemic.

The review provides the food industry with information about sanitizers and disinfectants with virucidal and inhibitory activity against SARS-CoV-2 or surrogates on food contact surfaces, liquids, aerosols and skin. The integration of the recommended disinfectants and infection-prevention approaches would prevent SARS-CoV-2 dissemination in food production, manufacturing and retail facilities and among personnel.

## Author Contributions

GD designed, performed literature review and contributed to the writing of the manuscript, and figure design. LD performed literature review and contributed to the writing of the manuscript and drafting of the table. AM and IO performed literature review and contributed to the writing of the manuscript. AT performed figure design, literature review and contributed to the writing of the manuscript. KB conducted literature review, writing of the manuscript and final review. CG assisted in the literature and final review. All authors contributed to the article and approved the submitted version.

## Conflict of Interest

The authors declare that the research was conducted in the absence of any commercial or financial relationships that could be construed as a potential conflict of interest.
